# Arrhythmia Identification with Two-Lead Electrocardiograms Using Artificial Neural Networks and Support Vector Machines for a Portable ECG Monitor System

**DOI:** 10.3390/s130100813

**Published:** 2013-01-09

**Authors:** Shing-Hong Liu, Da-Chuan Cheng, Chih-Ming Lin

**Affiliations:** 1 Department of Computer Science and Information Engineering, Chaoyang University of Technology, Taichung 41349, Taiwan; E-Mails: shliu@cyut.edu.tw (S.-H.L.); chihminglin800101@gmail.com (C.-M.L.); 2 Department of Biomedical Imaging and Radiological Science, China Medical University, Taichung 40402, Taiwan

## Abstract

An automatic configuration that can detect the position of R-waves, classify the normal sinus rhythm (NSR) and other four arrhythmic types from the continuous ECG signals obtained from the MIT-BIH arrhythmia database is proposed. In this configuration, a support vector machine (SVM) was used to detect and mark the ECG heartbeats with raw signals and differential signals of a lead ECG. An algorithm based on the extracted markers segments waveforms of Lead II and V1 of the ECG as the pattern classification features. A self-constructing neural fuzzy inference network (SoNFIN) was used to classify NSR and four arrhythmia types, including premature ventricular contraction (PVC), premature atrium contraction (PAC), left bundle branch block (LBBB), and right bundle branch block (RBBB). In a real scenario, the classification results show the accuracy achieved is 96.4%. This performance is suitable for a portable ECG monitor system for home care purposes.

## 1. Introduction

Telemedicine has been widely studied recently. In past research, allowing congestive heart failure patients to monitor their condition at home offered great economic advantages. Electrocardiograms (ECGs) are an important tool that provide useful information about the functional status of the heart. An automated method that accurately diagnoses cardiac diseases through the analysis of ECG signals is critical in healthcare [1], especially for real-time processing. Past research has addressed the problems of heart rate detection and classification of cardiac rhythms. The heart rate signal detects the QRS wave of the ECG and calculates inter-beat intervals [2–9]. The classification of cardiac rhythms is based on the detection of the different types of arrhythmia from the ECG waveforms [10–13].

However, ECG signals have coupling noises, due to factors such as 50/60 Hz power line signals, the baseline drift caused by patient breathing, bad electrodes, improper electrode location, or electromyograms. These noises result in false QRS wave detections. Thus, some studies have compared the robust performance of different algorithms for QRS wave detection [2]. Widrow *et al.* applied the adaptive filter to reduce noises that resulted from 60 Hz power lines and baseline drift, and then detect the QRS wave [14]. Pan and Tompkins designed a digital filter to reduce the noise and used a dynamic threshold to detect the QRS wave [4]. Trahanias used the mathematical morphology of the QRS complex to detect heart rates [5]. Chang used the ensemble empirical model decomposition to reduce noises in arrhythmia ECGs [15]. Fan used approximate entropy (ApEn) and Lempel-Ziv complexity as a nonlinear quantification to measure the depth of anaesthesia [16]. In these studies, the normal sinus ECG signal added different noise types and energy was used to evaluate the performance of these algorithms. Several researchers have extracted the features of ECG waveforms to detect the QRS complexes based on the arrhythmia database. Li *et al.* proposed the wavelet transforms method for detecting the QRS complex from high P or T waves, noise, and baseline drift [6]. Yeh and Wang proposed the difference operation method to detect the QRS complex waves [8]. Mehta and Lingayat used the support vector machine (SVM) method to detect the QRS complexes from a 12-leads ECG [9]. They also used the K-mean algorithm for the detection of QRS complexes in ECG signals [17].

Arrhythmia can be defined as either an irregular single heartbeat or a group of heartbeats. Some classification techniques are based on the ECG beat-by-beat classification with each beat being classified into several different arrhythmic beat types. These include artificial neural networks [11], fuzzy neural networks [18], Hermite functions combined with self-organizing maps [19], and wavelet analysis combined with radial basis function neural networks [20]. In these methods, the ECG waveform of each beat was picked up manually and different features were extracted to classify the arrhythmic types. Tsipouras *et al.* used the RR-interval signal to classify certain types of arrhythmia based on a group of heartbeats [12]. All the above methods have high classification accuracies that were obtained based on the complete morphology of the ECG or the correct RR-interval that was detected manually.

In this study, we propose an automatic configuration integrating digital signal processing and an artificial intelligence method to detect the position of heartbeats and recognize these heartbeats as belonging to the normal sinus rhythm (NSR) or four arrhythmic types. The four arrhythmic types are premature ventricular contraction (PVC), premature atrium contraction (PAC), left bundle branch block (LBBB), and right bundle branch block (RBBB). ECG signals are provided by the MIT-BIH Arrhythmia Database [21]. This automatic configuration had three steps, as follows:

1. The Lead II signals were normalized and filtered to reduce the coupled noise (Section 2.2).
2. The positions of QRS-complexes in Lead II were detected and marked via a well-trained SVM. Two waveforms of each heartbeat in Lead II and V1 were individually extracted according the markers in Lead II (Section 2.3).
3. The extracted waveform was used as a feature to recognize the arrhythmic type of a heartbeat. In this configuration, a self-constructing neural fuzzy inference network (SoNFIN) was used to recognize the arrhythmic type of the heartbeat using the raw Lead II and V1 signals (Section 2.4).

Moreover, the heartbeat detection accuracy has been increased by the SoNFIN classification results.

## 2. Experimental Section

**Figure 1.**
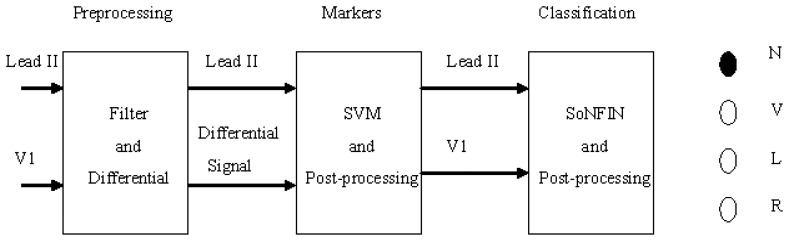
Stages of an automatic classifying system.

Figure 1 shows the schematic of this study. Two-lead ECG signals, Lead II and V1, are the inputs which are processed by digital filters to reduce the coupled noise. The filtered Lead II signal was differentiated to enhance the QRS complex. Lead II and its differential signal are used to mark the heartbeats (QRS-complex) with the SVM. Some redundant markers caused by the coupled noise were deleted by a postprocessor. According the marker, two segment waveforms containing the same QRS complex were extracted from the Lead II and V1 signals, individually. The SoNFIN used these waveforms as inputs to recognize the heartbeat type. The SVM used these markers to identify RR-intervals. All proposed algorithms for detection and classification of ECG signals were implemented on the MATLAB platform.

### 2.1. Database

The MIT-BIH Arrhythmia Database includes 48 ECG recordings, each of 30 min length, with a total of 109,000 R-R intervals. Each file has two-lead signals, Lead II and V1, V2, V4, or V5. The sampling rate was 360 Hz and it is digitized in 11 bits that ranged from 0 to 10 mV. In this study, since we only focus on the Lead II and V1 signals for pre-processing, 33 of the 48 files were selected to test the performance of SVM and SoNFIN. Each file gathered five-minute of data that only had NSR, PVC, PAC, LBBB, and RBBB signals. Table 1 shows the file number and the beat type, with a total of 12,776 beats.

### 2.2. Filtering and Normalization

A finite impulse response low-pass filter was used to reduce the interference of high frequency noise. The low-pass filter had order 10 and its cutoff frequency was 40 Hz. An adaptive filter was used to remove baseline wander when the reference input was constant [22]. The notch filter had a zero response at DC with a bandwidth that ranged from 0 to 0.5 Hz. Since the differential ECG signal had larger values in the QRS region than the non-QRS region, it was used as a feature to mark heartbeats. The raw ECG (Lead II and V1) signals and differential signals were normalized as follows:

(1) $$\text{Output}\_\text{data}=2\times \frac{\text{input}\_\text{data}-\min}{\max-\min}-1$$

where “min” is the minimal value and “max” is the maximal value.

### 2.3. QR-Complexes Detection and Waveform Extraction

This section gives a brief description on SVM for the two-class problem and introduces the training phase for the SVM. The goal of this process is to use SVM to mark QRS complexes using the normalized Lead II signals and its differential signals. After the test phase, post-processing was used to delete or merge the redundant markers caused by the coupled noise.

#### 2.3.1. Support Vector Machine (SVM)

SVM is a new learning system paradigm that has been widely used for solving supervised classification problems due to its generalization ability [23]. In essence, SVM classifies the maximum margin for the training data with a separating hyperplane that can be formulated as a quadratic optimization problem in feature space. The subsets of patterns closest to the decision boundary are called support vectors. Considering a linearly separable dataset {*X̅*, *D_i_*}, where *X̅* is the input pattern for the i-th example and *D_i_* is the corresponding desired output (1, or −1), a hyperplane is found as the decision surface. It can be written as follows:

(2) $$\begin{array}{l} W^{T}{\bar{X}}_{i}+b\geq 0\;\text{then},D_{i}=1\\ W^{T}{\bar{X}}_{i}+b<0\;\text{then},D_{i}=-1 \end{array}$$

where *W* is the coefficients' vector of the hyperplane function. The margin between the hyperplane and the nearest point is maximized and is considered a quadratic optimization problem:

(3) $$\min\frac{1}{2}(W^{T}W)$$

(4) $$\text{subject to}\;D_{i}(W^{T}{\bar{X}}_{i}+b)\geq 1$$

When *W* and *b* are rescaled, the point nearest to the hyperplane has a distance of $\overset{1}{\;}/\underset{\left\|W\right\|}{\;}$ [24]. Using Lagrange multipliers *α* ≥ 0 and the Kuhan-Tuker theorem, the solution is given by:

(5) $$W=\sum D_{i}\alpha_{i}{\bar{X}}_{i}$$

Only a small fraction of the *α_i_* coefficients is nonzero. The corresponding pairs of *X̅* are known as support vectors and they define the decision boundary. All other input patterns multiplied with zero *α_i_* values are rendered irrelevant. The hyperplane decision function for the input pattern vector *X̅* can be written as follows:

(6) $$f(x)=\mathrm{sgn}(\sum D_{i}\alpha_{i}({\bar{X}}_{i}^{T}{\bar{X}}_{i})+b)$$

By replacing the inner product $\left({\bar{X}}_{i}^{T}{\bar{X}}_{i}\right)$ with the kernel function *K*(*x*, *x_i_*), the input patterns are mapped to a higher dimensional space [24]. In this higher dimension, a separating hyperplane is constructed to maximize the margin.

#### 2.3.2. Training Phase of SVM

We trained SVM [25] to detect the QRS complexes for the positions of heartbeats. In this study, the Gaussian radial basis function was used to construct the kernel function as follows:

(7) $$K(x,x_{i})=\exp(-\gamma {\left\|x-x_{i}\right\|}^{2})$$

where *γ* = 2.5 is viewed as radial size. In order to have the best result, we trained the SVM for different *C* values. The best result was found when *C* = 200 in the training phase.

In the training process, the input features of SVM were the normalized Lead II and its differential signals. In each subject, we extracted only one cycle ECG waveform for each arrhythmic type and a NSR as the input. In the extracting range, the Lead II was used as the reference signal where the R-wave was assigned as the center point of a cycle waveform. According to Table 1, NSR had 27 files, PVC had 18 files, LBBB had four files, and RBBB had four files. Since the PAC waveforms were normal, none of them was selected for training. We individually extracted one cycle waveforms from these files that were not included in the five-minute test data for training purposes. Notably the test data (total 12,776 beats) were much larger than the training data. In total only 53 heartbeats (NSR had 27 beats; PVC had 18 beats; RBBB had four beats; and LBBB had four beats) were collected for training.

#### 2.3.3. Test and Post-Processing Phases of SVM

In the test phase, each file was extracted to provide five-minute data as the test instance that had been preprocessed (Section 2.2). There were 33 subjects and total of 12,776 heartbeats for testing. Normally the data length of the QRS complex contains at least 60 ms. Thus, if a marker duration (RR-interval) was less than 10 points (sampling rate: 360 Hz), this marker will be considered as a redundant marker caused by the couple noise and will be deleted. Moreover, if the distance between two neighbor markers was less than 5 points then these two markers will be merged as one marker.

### 2.4. Arrhythmia Classification

In this section, we used the SoNFIN as the classifier to recognize each ECG heartbeat type. Beats from the ECG have been marked by SVM may include mistaken beats. This was because SoNFIN belongs to the Takagi-Sugeno-Kang (TSK) model. We eliminated some mistaken markers that did not belong to the heartbeats according to the output value of SoNFIN.

#### 2.4.1. Self-Constructing Neural Fuzzy Inference Network (SoNFIN)

**Figure 2.**
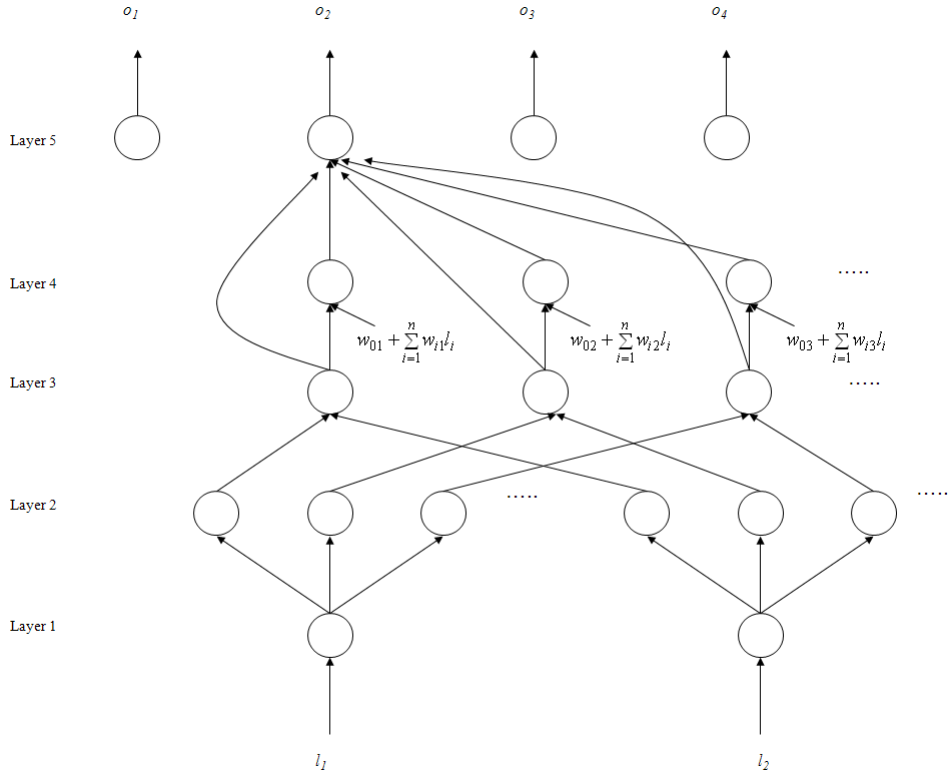
Structure of the SoNFIN.

The SoNFIN is a general connectionist model of a fuzzy inference system with a structure shown in Figure 2 [26]. This five-layer network realizes a fuzzy model of the following form:

$$\begin{array}{c} \text{Rule}\;j:\text{If}\;l_{i}\;\text{is}\;A_{1j}\;\text{and}\cdots \text{and}\;l_{n}\;\text{is}\;A_{nj}\\ \text{Then}\;z_{j}\;\text{is}\;w_{0j}+\sum_{i=1}^{n}w_{ij}l_{j} \end{array}$$

where *l_i_* is the input variable, *z_j_* is the output variable, *A_ij_* is a fuzzy set, and $w_{0j}+\overset{n}{\underset{i=1}{\text{∑}}}w_{ij}l_{i}$ is the traditional TSK model. The five layers are described below.

**Layer 1:** No computation is performed in this layer. Each node in this layer corresponds to one input variable. Only transmitted input values are forwarded to the next layer directly:
  (8) $$u_{i}^{\left(1\right)}=l_{i}$$
**Layer 2:** For fuzzy set *A_ij_*, a Gaussian membership function is used to describe the degree $u_{ij}^{\left(2\right)}$ that the input variable *l_j_* belongs to the *i*-th fuzzy set. Its mathematical function is defined as follows:
  (9) $$u_{ij}^{\left(2\right)}=\exp(\frac{-{\left[u_{i}^{\left(1\right)}-m_{ij}\right]}^{2}}{\sigma_{ij}^{2}})$$
  where *m_ij_* and *σ_ij_* are the center and width of the membership function, respectively. This function is implemented by each node.
**Layer 3:** A node in this layer represents one fuzzy logic rule and performs precondition matching of a rule. Here we use the following product operation for each Layer-3 node:
  (10) $$u_{j}^{\left(3\right)}=\prod_{i}u_{ij}^{\left(2\right)}$$
**Layer 4:** Nodes in this layer are called consequent nodes. Each node is linked to Layer-3 output, and the linear association of the weight in this layer is as follows:
  (11) $$u_{j}^{\left(4\right)}=u_{j}^{\left(3\right)}(w_{0j}+\sum_{i=1}^{n}w_{ij}l_{i})$$
**Layer 5:** Each node in this layer corresponds to one output variable. The node integrates all the actions recommended by Layer 5 and acts as a defuzzifier with:
  (12) $$o=u^{\left(5\right)}=\frac{\overset{p}{\underset{j=1}{\text{∑}}}u_{j}^{\left(4\right)}}{\overset{p}{\underset{j=1}{\text{∑}}}u_{j}^{\left(3\right)}}=\frac{\overset{p}{\underset{j=1}{\text{∑}}}u_{j}^{\left(3\right)}(w_{0j}+\overset{n}{\underset{i=1}{\text{∑}}}w_{ij}l_{i})}{\overset{p}{\underset{j=1}{\text{∑}}}u_{j}^{\left(3\right)}}$$

The number of network outputs is equal to the number of classes to be recognized (four in this study). The desired outputs, *d*, were (1, −1, −1, −1), (−1, 1, −1, −1), (−1, −1, 1, −1), and (−1, −1, −1, 1).

Two types of training (structure and parameter training) were used concurrently to construct the SoNFIN. Initially there were no rules in the SoNFIN, with all rules constructed by online structure training. For the training structure, a predefined threshold, *H*, was used as a criterion for the generation of fuzzy rules. When the maximum $u_{j}^{\left(3\right)}$ was below to *H* for every rule, a new rule was generated. Therefore, more rules were generated for a larger value of *H*. The initial width of each generated Gaussian fuzzy set was decided by a predefined constant σ.

To train the parameters the objective was to minimize the error function:

(13) $$V_{\text{error}}=\sum_{\text{i}=1}^{4}{\left(d_{i}-o_{i}\right)}^{2}$$

The consequent part parameters were tuned by the recursive least-squares algorithm. The fuzzy-set parameters were tuned by a gradient-descent algorithm. The details of the training algorithm may be found elsewhere [26].

#### 2.4.2. Training and Test Phases of SoNFIN

The input features of SoNFIN were normalized Lead II and V1 waveforms. Since the length of QRS complex is about 150 ms, the digitized segment is around 100 points. The R-wave is assigned as the middle point, and V1 is extracted in the same section. Therefore, the dimension of input vector is 200 (for Lead II and V1). In the training phase, for each heartbeat type (NSR, PVC, LBBB, and RBBB) 26 patterns were extracted from 33 files that did not belong to the five-minute test data. In tuning the parameters of SoNFIN, we have done our best to get the optimal parameters with *H* = 0.1, and σ = 0.6. Training was performed in 1,000 iterations. The training rates of the consequent and fuzzy-set parts were 0.01 and 0.05, respectively.

In the test phase, an automated algorithm was designed to classify each heartbeat type of 33 files extracted from five-minute data. The procedure of this algorithm is described as below. First, we used the positive edge of markers of Lead II that had been determined in Section 2.3.3 to be the reference point. Second, starting from this reference point, we forwardly found a minimum point within 100 points in Lead II. According to this minimum point, a maximum point was looked up within 50 points, backwardly. Third, the maximum point (R-wave) was used as the middle point to extract 100 points. V1 was extracted in the same section.

In the test results, the real output of the SoNFIN was denoted by (*o_1_*, *o_2_*, *o_3_*, *o_4_*). The output node with the maximum value was then found. If *o_i_* is the maximum value, then the unknown beat was recognized as belonging to class *i*. However, some mistaken markers in the ECG signal were fed into SoNFIN to identify class. The output value of the mistaken marker was higher than the truthful heartbeat. Thus, we designed a threshold for the output to delete mistaken markers.

## 3. Results and Discussion

**Figure 3.**
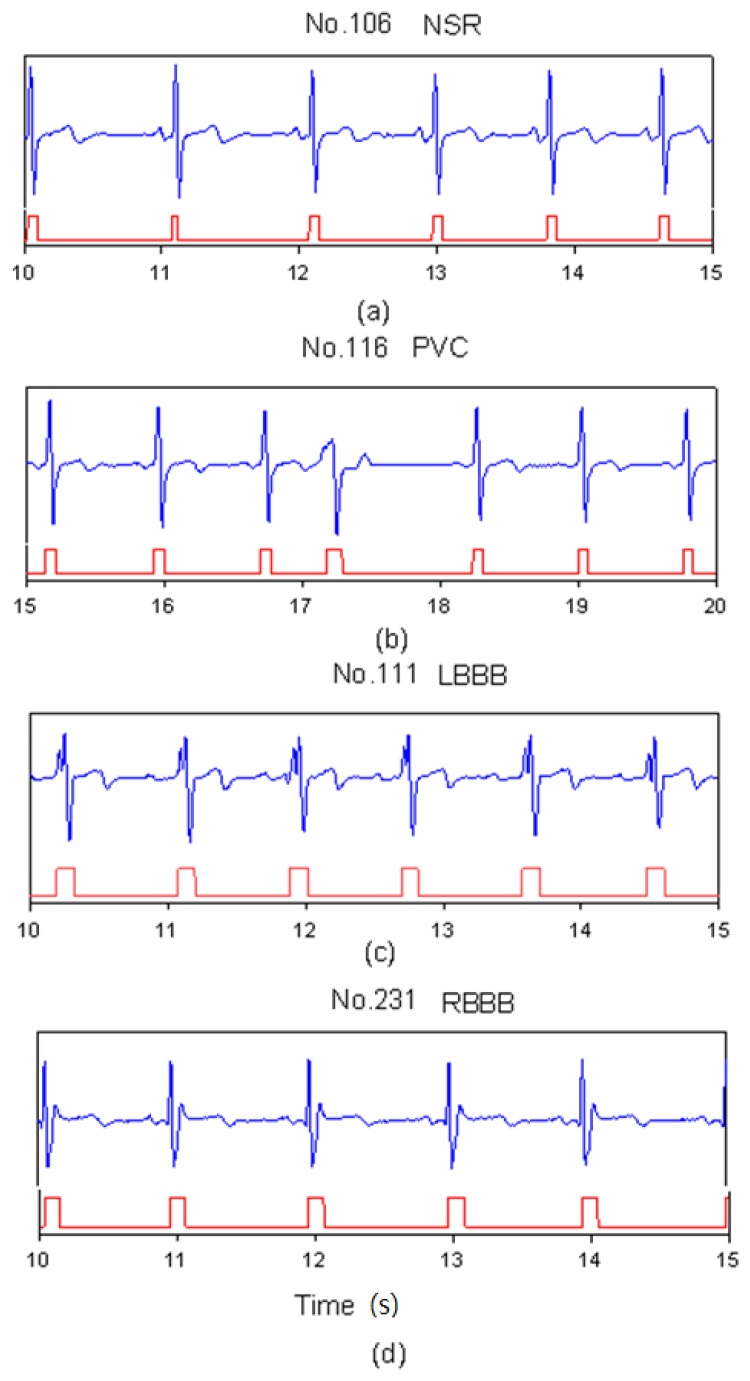
SVM marks the heartbeats of ECG, (**a**) NSR beats for file No. 106, (**b**) PVC beats for file No. 116, (**c**) LBBB beats for file No. 111, and (**d**) RBBB beats for file No. 231.

In heartbeat marking results, Figure 3 shows the markers of ECG heartbeats for four types (NSR, PVC, LBBB, and RBBB). In Figure 3(a), since the NSR has a standard QRS complex, the range of the marker includes a full QRS complex. In Figure 3(b), the PVC beat has an inverse QRS complex. The marker only happens in the position of the positive peak. For LBBB case, the Q-wave was lost and there were two neighboring positive peaks in one beat as shown in Figure 3(c). Therefore, post-processing did the merging function for this situation. Table 2 shows the number of correct markers (TP), missing markers (FN), and mistaken markers (FP) in all files. There were a total of 22 missing markers and 572 mistaken markers from all files. The FN ratio was 0.17%, the FP ratio was 4.48%.

**Figure 4.**
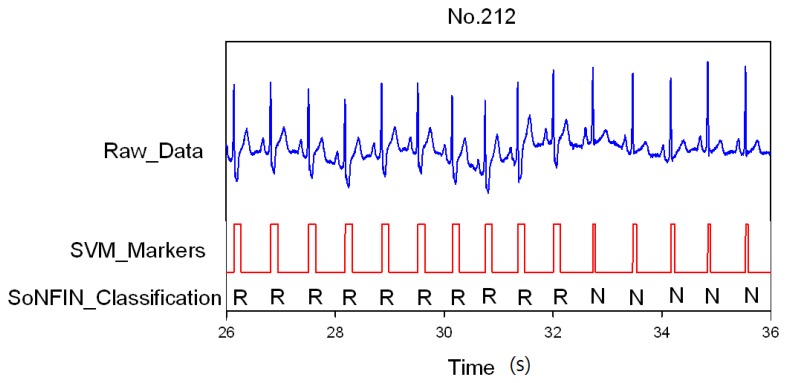
SVM marks the heartbeats of ECG, and then SoNFIN classifies the RBBB and NSR beats for file No. 212, in which “R” and “N” denotes RBBB and NSR, respectively.

**Figure 5.**
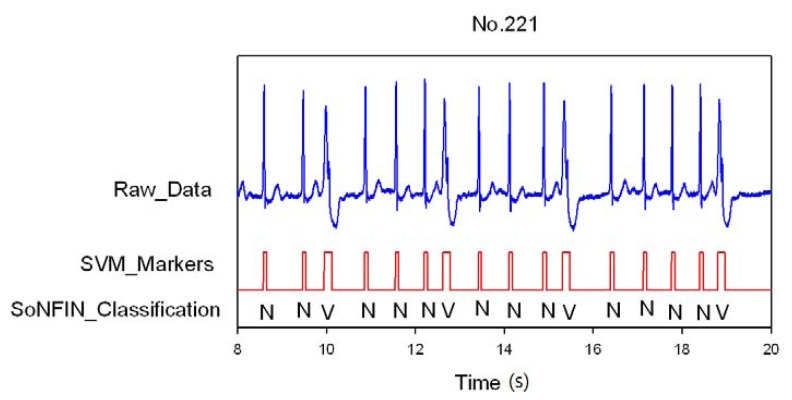
SVM marks the heartbeats of ECG and then SoNFIN classifies the NSR and PVC beats for file No. 221, in which “N” and “V” denotes NSR and PVC, respectively.

**Figure 6.**
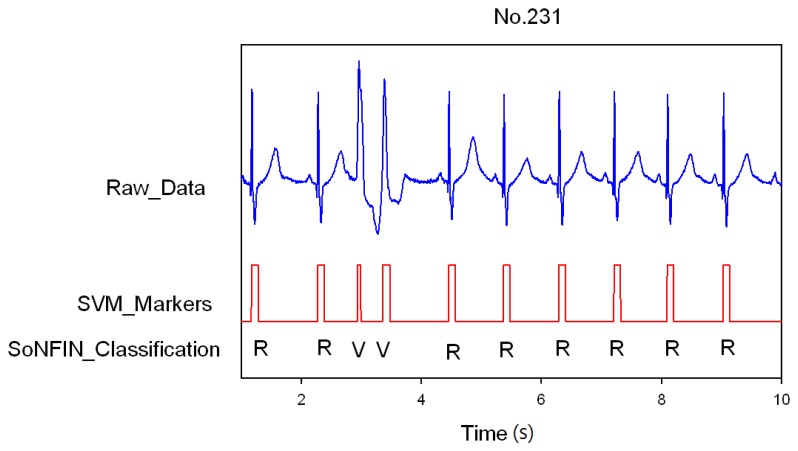
SVM marks the heartbeats of ECG, and then SoNFIN classifies continuous two PVC beats happening in the RBBB beats for file No. 231.

Classification results of the SoNFIN have two conditions. The first condition doesn't care about the the FN and FP of the heartbeat detection. Figure 4 shows the marked and classified results for the subject of file number 212. It has continuous LBBB beats and NSR beats. In Figure 5, the subject in file number 221 has discrete PVC beats in the continuous NSR beats. Figure 6 shows continuous PVC beats in the RBBB beats for the subject in file number 231. The classified test results are shown in Table 3, where each cell contains the raw number of exemples classified for the corresponding combination of desired and actual outputs. In this table, 9,189 beats were correctly classified to NSR, 684 beats were correctly classified to PVC, 1,287 beats were correctly classified to RBBB, and 1,419 beats were correctly classified to LBBB. The classification performances of SoNFIN were examined based on sensitivity, specificity, and total classification accuracy. The sensitivity was the number of TP divided by the number of actual positive cases. Specificity was the number of TN divided by the number of actual negative cases.

Total classification accuracy was the number of correct decisions divided by the total number of cases. Table 3 showed the sensitivity, specificity, and averaged accuracy. Under these conditions, the average accuracy was 98.8%. In a real scenario, the FN and FP of the heartbeat detection must happen. Therefore, the second condition was to classify all marked waves including 572 false heartbeats. The output value of the false heartbeat would be higher than that of a truthful heartbeat. Thus, we designed a threshold, 2.5, to determine the false heartbeats belonging to the noise, as shown in Table 4. The false heartbeats were reduced to 301. The classification accuracy is only 96.4%. Moreover, the specificity of false heartbeat is 100% in heartbeat classification, and the FP also decreased to 2.4% in heartbeat detection.

## 4. Conclusions

The digital processing method for determining heartbeats in real time was to enhance the QRS complex of a one-lead ECG signal with a differential method and set a threshold to find the position of the R-wave [2,4,27,28]. In enhancing QRS complex waves, non-differential methods like Hilber transform [29], wavelet transform [6], moving averaging incorporating with wavelet [30], principle component analysis [31], and Karhunen-Loève transform [32] were used. Recently, Mehta *et al.* used the SVM method [9] and K-mean algorithm [17] for 12 lead ECG signals to detect heartbeats. SVM found a hyperplane for separating the maximum margin of the classified set. Mehta and Lingayat used 1,488 heartbeats to evaluate the performance of their algorithm [9]. Since the SVM method easily marked the larger P or T waves as the heartbeats, Mehta's method had 24 mistaken markers and four missing markers. Moreover, they used 12 lead ECG signals to detect the heartbeats, which was easier than using a one-lead ECG signal. The measurement of 12 lead ECG signals was not suitable for a real-time or portable system.

The significance of our study can be summarized as follows: we only used one-lead ECG, Lead II and its differential signal, as the input to mark QRS complexes. The proposed scheme was suitable for a portable system. A total of 12,776 heartbeats were used to test the performance of our scheme. The hyperplane of SVM in the two dimensions worked as a threshold to detect the QRS complex. Therefore, the deletion and merging processes were used to reduce the mistaken markers that occurred from noises or cardiac diseases. The results showed that the sensitivity of our method is 99.8%, the FN ratio is 0.17%, and the FP ratio is 4.48%. When all marked waves were classified by SoNFIN, the larger P waves, T waves, or noises could be filtered. Thus, the number of mistaken markers decreased to 301. The FP decreased to 2.4%, and the accuracy was increased to 97.5%. Table 5 displays the comparison of the various QRS detection algorithms with the same input method. Although some previous studies had shown better performance, as shown in Table 5 [4,5,7–9,17,28,32], however, as we emphasized, our method used the raw signal and the differential signal of only one-lead ECG as input. This major difference was that we have successfully developed a portable ECG monitoring system for patient use at home.

**Figure 7.**
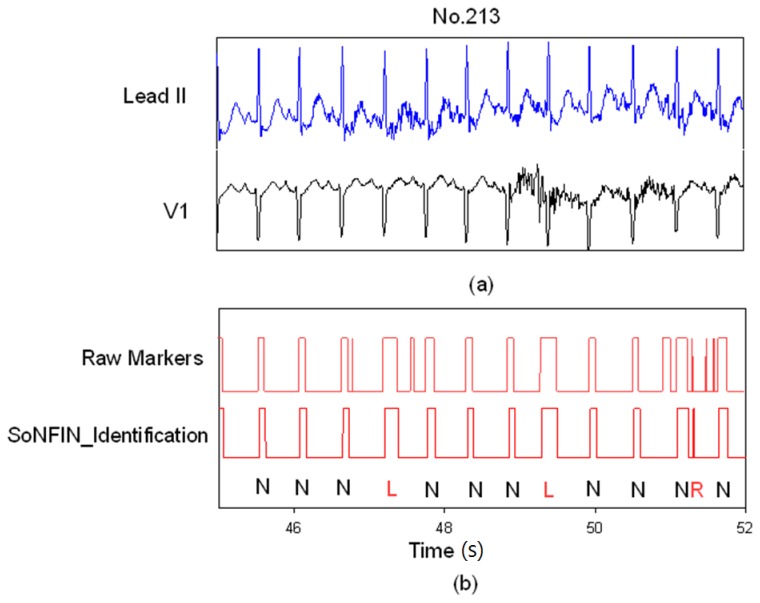
SoNFIN filters the mistaken markers for file No. 231, (**a**) the raw Lead II and V1 signals; (**b**) the raw markers by SVM and the filtered Markers by SoNFIN.

Figure 7 demonstrates how the SoNFIN increased the accuracy of the SVM heartbeat recognition. Figure 7(a) shows the filtered and normalized Lead II and V1 signals from 47 s to 51 s for the subject of file 213.

Since the filtered ECG signals have some noises, there are five mistaken markers in the upper Figure 7(b). These mistaken markers were deleted (four mistaken markers) via the SoNFIN as shown in the lower Figure 7(b). The residual mistaken marker was classified as the RBBB beat.

Since the heart is an elastic and relative solid organ, clinical diagnosis needs 12 lead ECG signals to identify different cardiac diseases. Therefore, the less lead-signal numbers there are, the less classification types it receives. Therefore, the recognition and classification are more difficult.

In conclusion, we have proposed an automatic scheme integrating the SVM and SoNFIN, and used only one-lead ECG (Lead II) to detect the heartbeats. Two-lead ECG (Lead II and V1) were used to identify the type of arrhythmia. In a real scenario, the average accuracy for the arrhythmia identification was 96.4%. This accuracy is clinically acceptable for a portable monitor system for only two-lead ECG input. The proposed configuration was applicable for homecare or long-term automatic ambulatory cardiac diagnosis.

## Figures and Tables

**Table 1.** The selected 33 files and the number of each arrhythmia type.

	N	V	R	L	A	total
105	401	15	0	0	0	416
106	312	2	0	0	0	314
108	275	5	0	0	2	282
109	0	7	0	411	2	420
111	0	0	0	343	0	343
112	428	0	0	0	0	428
113	288	0	0	0	1	289
115	316	0	0	0	0	316
116	384	11	0	0	0	395
118	0	3	347	0	11	361
119	245	80	0	0	0	325
121	301	0	0	0	0	301
122	421	0	0	0	0	421
201	441	0	0	0	0	441
202	261	4	0	0	0	265
205	449	3	0	0	0	452
207	0	0	0	349	0	349
208	242	245	0	0	0	487
209	365	0	0	0	178	543
212	140	0	319	0	0	459
213	501	48	0	0	0	549
214	0	33	0	346	1	380
219	364	15	0	0	0	379
220	352	0	0	0	1	353
221	327	80	0	0	0	407
222	366	0	0	0	0	366
223	390	16	0	0	0	406
228	312	18	0	0	0	330
230	392	0	0	0	0	392
231	13	1	287	0	0	301
232	0	0	330	0	0	330
233	372	138	0	0	4	514
234	462	0	0	0	0	462
Total	9,120	724	1,283	1,449	200	12,776

N is NSR, V is PVC, L is LBBB, R is RBBB, and A is PAC. The first column denotes the file number.

**Table 2.** Results of marker detection using SVM.

No.	TP	FN	FP
105	416	0	24
106	312	2	26
108	282	0	37
109	419	1	10
111	343	0	186
112	428	0	81
113	289	0	0
115	316	0	0
116	395	0	4
118	361	0	11
119	325	0	12
121	301	0	20
122	421	0	0
201	441	0	0
202	265	0	1
205	452	0	0
207	349	0	13
208	480	7	11
209	541	2	52
212	455	4	9
213	548	1	4
214	376	4	8
219	378	1	9
220	353	0	0
221	407	0	0
222	366	0	5
223	406	0	0
228	330	0	1
230	392	0	0
231	301	0	41
232	330	0	3
233	514	0	4
234	462	0	0
Total	12,754	22	572

TP: true positive, FN: false negative, FP: false positive. We define TN = 0.

**Table 3.** Statistical values of the SoNFIN classification results of the test phase in first condition.

N		Estimate		Sensitivity	Specificity	Accuracy
		N	Non_N			
Real	N	9,189	107	98.8%	99.2%	98.9%
	Non_N	25	3,433			
V		Estimate		95.1%	99.4%	99.1%
		V	Non_V			
Real	V	684	35			
	Non_V	72	11,998			
R		Estimate		99.7%	99.8%	99.8%
		R	Non_R			
Real	R	1,287	3			
	Non_R	20	11,444			
L		Estimate		97.9%	99.4%	99.3%
		L	non_L			
Real	L	1,419	30			
	Non_L	58	11,247			
Averaged accuracy						98.8%

**Table 4.** Statistical values of the SoNFIN classification results of the test phase in second condition.

N		Estimate		Sensitivity	Specificity	Accuracy
		N	Non_N			
Real	N	9,189	107	98.8%	96.9%	98.2%
	Non_N	121	3,909			
V		Estimate		95.1%	98.1%	97.9%
		V	Non_V			
Real	V	684	35			
	Non_V	239	12,368			
R		Estimate		99.7%	99.7%	99.7%
		R	Non_R			
Real	R	1,287	3			
	Non_R	31	12,005			
L		Estimate		97.9%	99.2%	99.1%
		L	non_L			
Real	L	1,419	30			
	Non_L	85	11,792			
noise		Estimate		47.7%	100%	97.7%
		noise	Non_noise			
Real	n	271	301			
	Non_noise	0	12,754			
Averaged accuracy						96.4%

**Table 5.** Comparison with other QRS detection algorithms.

**Reference**	**Method**	**Accuracy (%)**
Proposed algorithm	SVM	97.5%
J. Pan, and W. J. Tompkins [4]	Dynamic threshold	99.3%
P. E. Trahanias [5]	Mathematical morphology	99.48%
F. Gritzali [7]	Length and energy transformation	99.6%
Y. -C. Yeh, and W. -J. Wanga [8]	Difference operation method	99.81
M. Adnane *et al.* [28]	Morphological features	99.64%
M. Paoletti and C. Marchesi [32]	Karhunen-Loève transform	99.15%
S. S. Mehta and N. S. Lingayat [9]	SVM	98.12%
S. S. Mehta *et al.* [17]	K-mean	98.66%
